# Prevalence of symptoms, comorbidities, fibrin amyloid microclots and platelet pathology in individuals with Long COVID/Post-Acute Sequelae of COVID-19 (PASC)

**DOI:** 10.1186/s12933-022-01579-5

**Published:** 2022-08-06

**Authors:** Etheresia Pretorius, Chantelle Venter, Gert Jacobus Laubscher, Maritha J Kotze, Sunday O. Oladejo, Liam R. Watson, Kanshu Rajaratnam, Bruce W. Watson, Douglas B. Kell

**Affiliations:** 1 Department of Physiological Sciences, Faculty of Science, Stellenbosch University, Private Bag X1, Matieland, Stellenbosch, 7602 South Africa; 2 Mediclinic Stellenbosch, Stellenbosch, 7600 South Africa; 3 Division of Chemical Pathology, Department of Pathology, National Health Laboratory Service, Tygerberg Hospital & Faculty of Medicine and Health Sciences, Stellenbosch University, Cape Town, 8000 South Africa; 4 Centre for AI Research, School for Data-Science & Computational Thinking, Stellenbosch University, Stellenbosch, 7600 South Africa; 5 Department of Biochemistry and Systems Biology, Faculty of Health and Life Sciences, Institute of Systems, Molecular and Integrative Biology, University of Liverpool, Liverpool, L69 7ZB UK; 6 The Novo Nordisk Foundation Centre for Biosustainability, Technical University of Denmark, Kemitorvet 200, 2800 Kgs Lyngby, Denmark

**Keywords:** Long COVID/PASC, Symptoms, Co-morbidities, Amyloid fibrin(ogen), Hyperactivated platelets, Fluorescence microscopy

## Abstract

**Background:**

Fibrin(ogen) amyloid microclots and platelet hyperactivation previously reported as a novel finding in South African patients with the coronavirus 2019 disease (COVID-19) and Long COVID/Post-Acute Sequelae of COVID-19 (PASC), might form a suitable set of foci for the clinical treatment of the symptoms of Long COVID/PASC. A Long COVID/PASC Registry was subsequently established as an online platform where patients can report Long COVID/PASC symptoms and previous comorbidities.

**Methods:**

In this study, we report on the comorbidities and persistent symptoms, using data obtained from 845 South African Long COVID/PASC patients. By using a previously published scoring system for fibrin amyloid microclots and platelet pathology, we also analysed blood samples from 80 patients, and report the presence of significant fibrin amyloid microclots and platelet pathology in all cases.

**Results:**

Hypertension, high cholesterol levels (dyslipidaemia), cardiovascular disease and type 2 diabetes mellitus (T2DM) were found to be the most important comorbidities. The gender balance (70% female) and the most commonly reported Long COVID/PASC symptoms (fatigue, brain fog, loss of concentration and forgetfulness, shortness of breath, as well as joint and muscle pains) were comparable to those reported elsewhere. These findings confirmed that our sample was not atypical. Microclot and platelet pathologies were associated with Long COVID/PASC symptoms that persisted after the recovery from acute COVID-19.

**Conclusions:**

Fibrin amyloid microclots that block capillaries and inhibit the transport of O_2_ to tissues, accompanied by platelet hyperactivation, provide a ready explanation for the symptoms of Long COVID/PASC. Removal and reversal of these underlying endotheliopathies provide an important treatment option that urgently warrants controlled clinical studies to determine efficacy in patients with a diversity of comorbidities impacting on SARS-CoV-2 infection and COVID-19 severity. We suggest that our platelet and clotting grading system provides a simple and cost-effective diagnostic method for early detection of Long COVID/PASC as a major determinant of effective treatment, including those focusing on reducing clot burden and platelet hyperactivation.

## Introduction

As approximately 30% of COVID-19 patients infected with the severe acute respiratory syndrome coronavirus 2 (SARS-CoV-2) continue, and in some cases begin, to suffer a variety of debilitating symptoms weeks or months after the acute phase of infection. The precise definition of this Long COVID/Post-Acute Sequelae of COVID-19 (PASC) (here referred to as Long COVID/PASC) is rather unclear and in some instances even vague. This is because most pathophysiological mechanisms involved have not yet been fully identified, and many different symptoms have been reported. The most frequently reported symptoms persist for 6 months or longer after acute infection [1]. COVID-19 survivors complain of recurring fatigue or muscle weakness, being out of breath, sleep difficulties, and suffer from anxiety or depression [2]. Symptoms noted in Long COVID/PASC patients show numerous similarities to those seen in chronic illnesses, including Myalgic Encephalomyelitis/Chronic Fatigue Syndrome (ME/CFS) [3–8], Postural Orthostatic Tachycardia Syndrome [9] and Mast Cell Activation Syndrome [1, 10]. In a large global survey of 3762 Long COVID/PASC patients from 56 countries it was found that nearly half still could not work full-time 6 months post-infection, due mainly to fatigue, post-exertional malaise, and cognitive dysfunction [11].

An important component of severe COVID-19 disease is virus-induced endothelialiitis. This leads to disruption of normal endothelial function, initiating a state of failing normal clotting physiology. Massively increased levels of von Willebrand Factor (VWF) lead to overwhelming platelet activation, as well as activation of the enzymatic (intrinsic) clotting pathway. We have previously found persistent circulating fibrin amyloid microclots, that are resistant to fibrinolysis, in samples from acute COVID-19 patients [12, 13]. We also, for the first time, reported on the minor effect of clotting in a patient with the Omicron variant [14]. Endothelial, microclot and platelet pathologies are also present in Long COVID/PASC patients [15, 16]. In a recent study we identified numerous dysregulated molecules in circulation that might cause or reflect the lingering symptoms for those individuals with Long COVID/PASC [15]. We used proteomics to study the proteins present in both digested supernatant and trapped persistent pellet deposits (after protein digestion via trypsin). Dysregulated molecules include the acute phase inflammatory molecule Serum Amyloid A (SAA) and α(2)-antiplasmin (α2AP). We had previously discovered that in many chronic diseases fibrinogen can clot into an amyloid form that is resistant to fibrinolysis, and that these fibrin amyloid (micro)clots could be detected with a fluorogenic amyloid stain [12, 17–24]. Thus, we used fluorescence microscopy to report large amyloid microclots and hyperactivated platelets present in blood samples from Long COVID/PASC; we also showed that these deposits are highly resistant to fibrinolysis [15, 25]. The plasmin-antiplasmin system plays a key role in blood coagulation and fibrinolysis [26]. Plasmin and α2AP are primarily responsible for a controlled and regulated dissolution of the fibrin polymers into soluble fragments such as d-dimer [26, 27]. We also developed a platelet and microclot grading system to classify platelet and microclot pathology [28]. The grading system should ideally be applied as part of a multi-pronged approach, which in addition to appropriate anticoagulation, may also include antiviral treatment to limit cell entry of SARS-CoV2.

Differentiation of platelet and microclot pathology due to Long COVID/PASC from cardiovascular disease (CVD), hypertension, hypercholesterolemia or diabetes as the main co-morbidities associated with SARS CoV-2 infection, is important in our search for ways to influence the underlying pathogenesis prophylactically. Many of the Long COVID/PASC symptoms that have been reported are related to symptoms that are cardio-pulmonary in nature. In this work we present results from a cohort of 845 patients who completed an online South African Long COVID/PASC registry. In parallel, blood samples of 80 patients who visited the clinical practice of our clinical co-author were collected to report on the presence of microclots and platelet pathology associated with persistent symptoms after recovery from acute COVID-19. Before contracting acute COVID-19, these patients did not suffer from fatigue and other symptoms that they subsequently reported, and which are typically associated with Long COVID/PASC. Thus, they were diagnosed as having Long COVID/PASC by means of eliminating all other common diseases, including heart failure.

## Materials and methods

### Ethical clearance

Ethics approval for the study was obtained from the Health Research Ethics Committee (HREC) of Stellenbosch University, South Africa, with references B21/03/001_COVID-19, project ID: #21911 (long COVID registry data) and N19/03/043, project ID #9521 (Long COVID blood collection). The experimental objectives, risks, and details were explained to volunteers and informed consent were obtained prior to blood collection. Strict compliance to ethical guidelines and principles of the Declaration of Helsinki, South African Guidelines for Good Clinical Practice, and Medical Research Council Ethical Guidelines for Research were kept for the duration of the study and for all research protocols.

### Data collection and analysis of patients who filled in the South African Long COVID/PASC registry

The South African Long COVID/PASC registry is an online platform where patients can self-report long COVID/PASC symptoms and previous comorbidities. Data were analysed for risk factors associated with developing Long COVID/PASC. All data were anonymised. The statistical analysis of the South African Long COVID registry data was carried out in a Jupyter notebook environment [29] and the Pandas library [30] was employed for data manipulation and statistical analysis. With the aid of an interactive python data library, Plotly (https://plot.ly), visualisation of the statistical analysis was drawn using Sankey plots.

We have also used lattices (a technique based in knowledge representation and artificial intelligence) to visually represent the data. Lattices for exploratory data-science and artificial intelligence are less common than other techniques, but often yield different insights and paths for further exploration [31]. An introduction to lattices in exploratory data-science is given in [32] among others. The “conexp” software package (freely available from conexp.sourceforge.net) was used for preparing the lattices in this paper. The data of the 845 participants in the cohort were condensed into a single matrix mapping comorbidities to symptoms, in preparation for drawing the lattices. The input was a comma-separated values (CSV) file containing one patient per row, with entries of 0 or 1 (absence or presence) in columns, where the comorbidities and symptoms appear as individual columns. First, the percentage prevalence for each symptom was calculated by traversing all rows; this was subsequently used as a threshold vector. Next, for each comorbidity, the comorbidity-implied percentage prevalence was calculated for each symptom, giving a matrix of comorbidity (rows) versus symptoms (columns) and percentage entries. Finally, in order to draw easily visualised lattices with 0 and 1 entries, this last matrix was normalised based on the initially calculated threshold vector.

### Blood sample collection from the cohort of 80 patients

Blood was drawn from 80 patients (35 females and 45 males); (mean/SD age 48 ± 16) who visited our clinical collaborator’s practice. Either a qualified phlebotomist or medical practitioner drew citrated blood into sample tubes (BD Vacutainer®, 369714), via venepuncture, adhering to standard sterile protocol. Whole blood (WB) was centrifuged at 3000×*g* for 15 min at room temperature and the supernatant platelet poor plasma (PPP) samples were collected and stored in 1.5 mL Eppendorf tubes at − 80 °C, until the analysis was performed. Haematocrit samples were analysed on the day of collection.

#### Long COVID/PASC diagnosis

Patients gave consent to study their blood samples, following clinical examination. Participants who filled in the South African Long COVID/PASC registry, gave consent on the platform for the team to use their de-identified data. Symptoms must have been new and persistent symptoms noted after acute COVID-19. Initial patient diagnosis of the 80 participants who visited our clinical collaborator’s practice (and who gave a blood sample), was the end result of exclusions, only after all other pathologies had been excluded. This was done by taking a history of previous symptoms (before and after acute COVID-19 infection), clinical examinations, and investigations including: full blood counts; N-terminal pro b-type natriuretic peptide (NTproBNP) levels (if raised it suggests cardiac damage); thyroid-stimulating hormone (TSH) and C-reactive protein levels. Lingering symptoms that can be ascribed to Long COVID/PASC were then assessed and included shortness of breath; recurring chest pain; lingering low oxygen levels; heart rate dysfunction (heart palpitations); constant fatigue (more than usual); joint and muscle pain; brain fog; lack of concentration; forgetfulness; sleep disturbances and digestive and kidney problems. These symptoms should have been persistent and new symptoms that were not present before acute COVID-19 infection and persistent for at least 2 months after recovery from acute (infective) COVID-19. This part of the examination was done only where the participants gave a blood sample.

#### Platelet pathology

Haematocrit samples of all 80 patients in the cohort were exposed to the two fluorescent markers, CD62P (PE-conjugated) (platelet surface P-selectin) (IM1759U, Beckman Coulter, Brea, CA, USA) and PAC-1 (FITC-conjugated) (340507, BD Biosciences, San Jose, CA, USA). CD62P is a marker for P-selectin that is either on the membrane of platelets or found inside them [13, 33]. PAC-1 identifies platelets through marking the glycoprotein IIb/IIIa (gpIIb/IIIa) on the platelet membrane. To study platelet pathology, 4 µL CD62P and 4 µL PAC-1 was added to 20 µL haematocrit, followed by incubation for 30 min (protected from light) at room temperature. The excitation wavelength band for PAC-1 was set at 450 to 488 nm and the emission at 499 to 529 nm and for the CD62P marker it was 540 nm to 570 nm and the emission 577 nm to 607 nm. Samples were viewed using a Zeiss Axio Observer 7 fluorescent microscope with a Plan-Apochromat 63x/1.4 Oil DIC M27 objective (Carl Zeiss Microscopy, Munich, Germany).

#### Platelet poor plasma (PPP) and the detection of amyloid fibrin(ogen) protein and anomalous microclotting

Microclot formation in PPP samples from all 80 Long COVID/PASC patients were analysed. These patients were diagnosed by our clinical collaborators, and they were not yet placed on any clinician-initiated treatment regimens. PPP were exposed to the fluorescent amyloid dye, Thioflavin T (ThT) (final concentration: 0,005mM) (Sigma-Aldrich, St. Louis, MO, USA) for 30 min (protected from light) at room temperature [20–23]. After incubation, 3 µL PPP was placed on a glass slide and covered with a coverslip. The excitation wavelength band for ThT was set at 450 nm to 488 nm and the emission at 499 nm to 529 nm and processed samples were viewed using a Zeiss Axio Observer 7 fluorescent microscope with a Plan-Apochromat 63x/1.4 Oil DIC M27 objective (Carl Zeiss Microscopy, Munich, Germany) [12, 13, 25].

## Results

### South African Long COVID/PASC registry

**Fig. 1:**
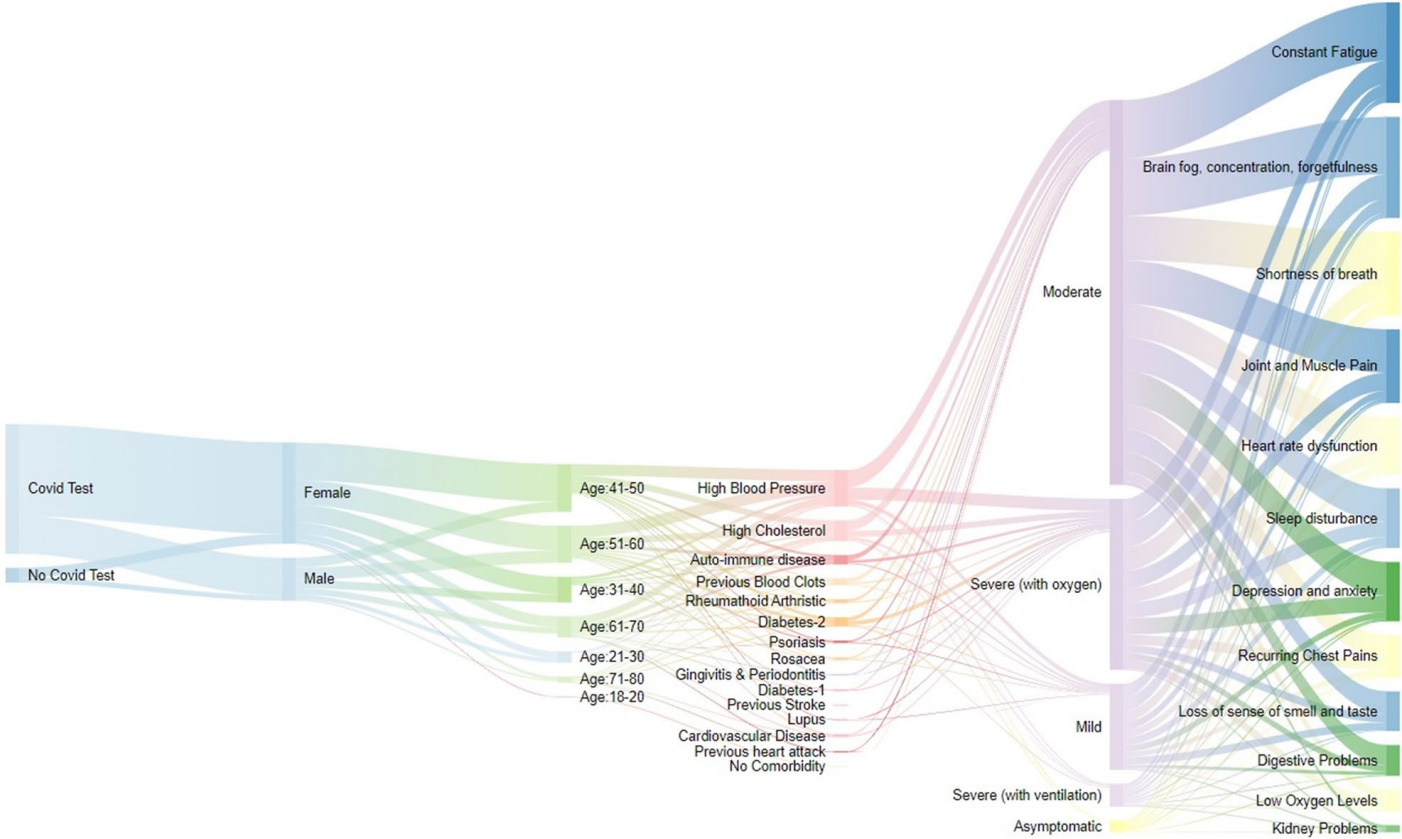
General overview of the population distribution of the South African Long COVID/PASC registry data as presented in a Sankey plot

**Fig. 2:**
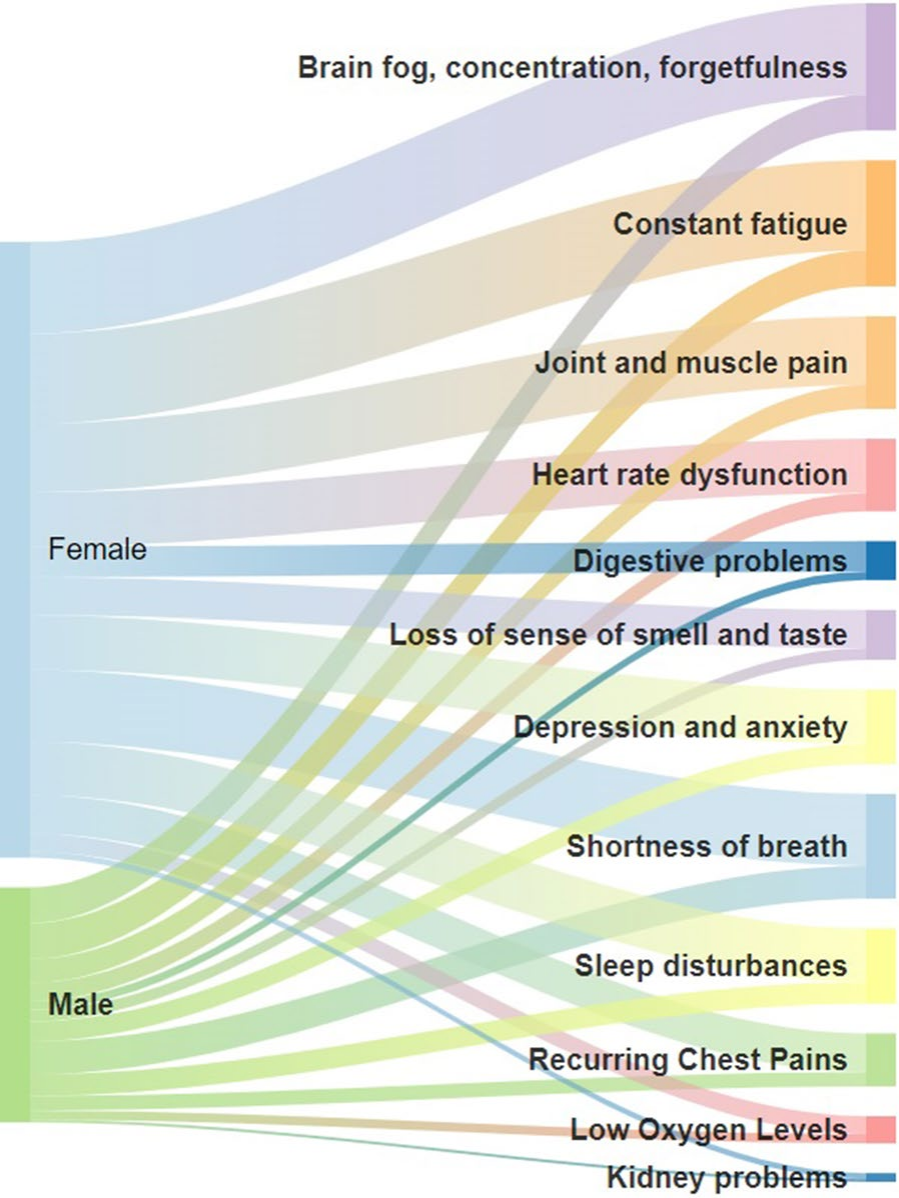
Gender versus Long COVID/PASC symptoms population distribution of the South African Long COVID registry data

**Fig. 3:**
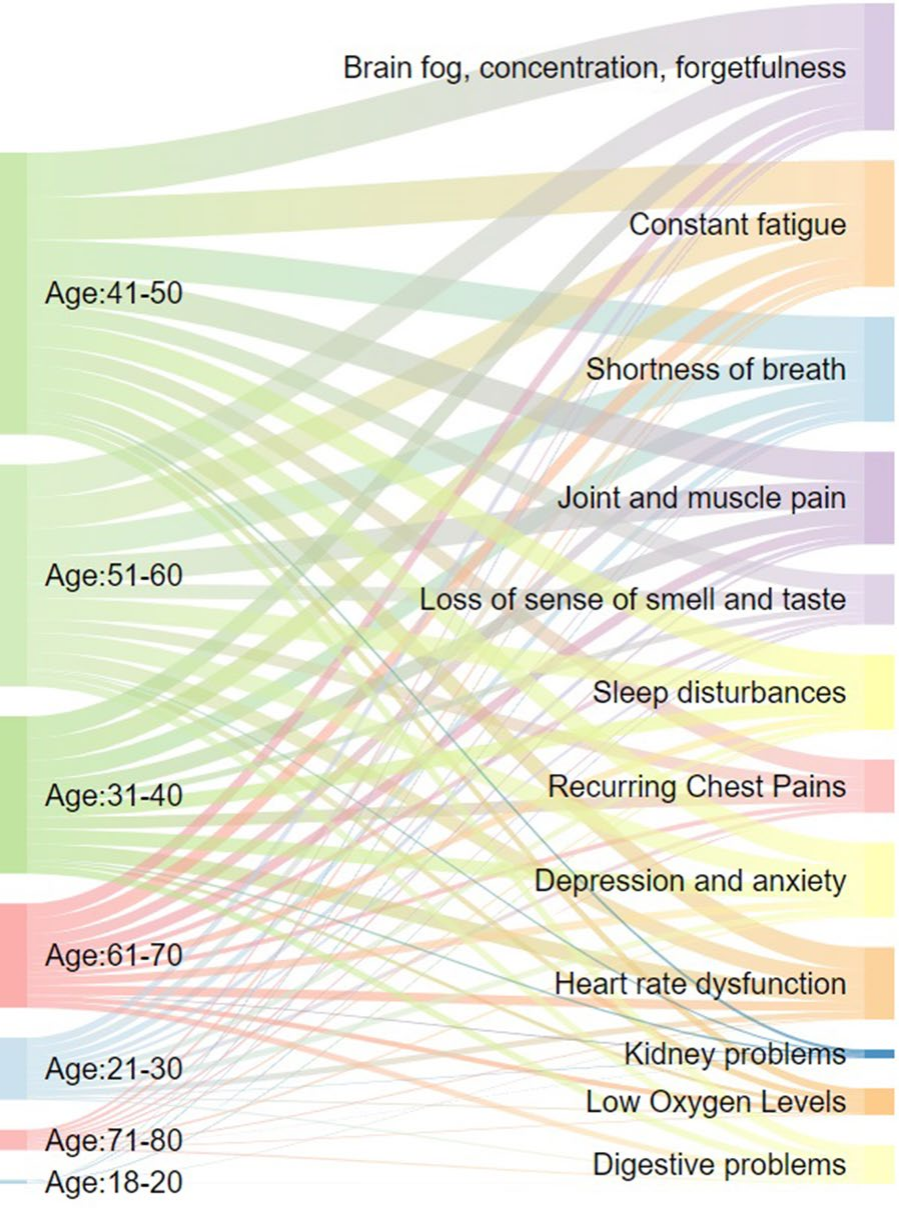
Age versus Long COVID/PASC symptoms participant distribution of the South African Long COVID/PASC registry data

**Fig. 4:**
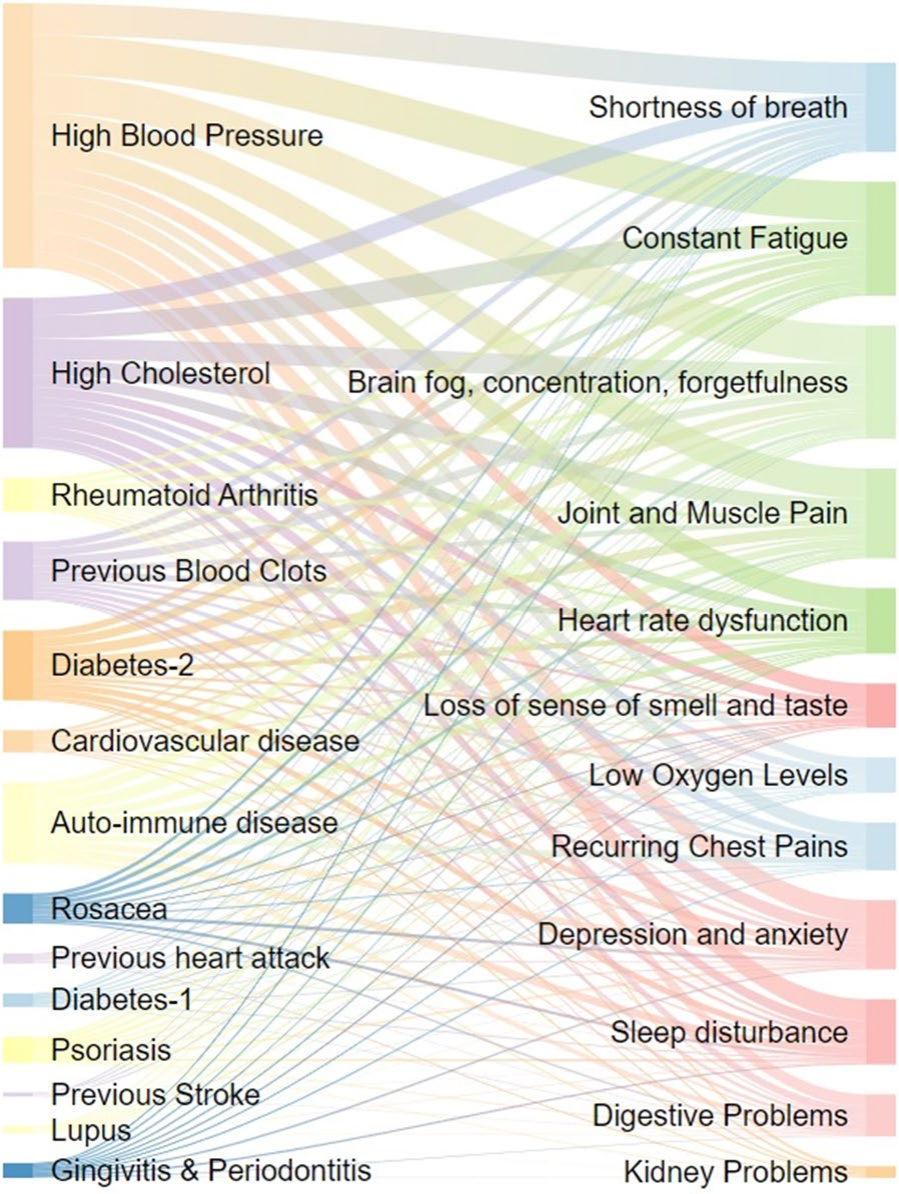
A Sankey plot showing participants comorbidities versus Long COVID/PASC symptoms population distribution of the South African Long COVID registry data

**Fig. 5:**
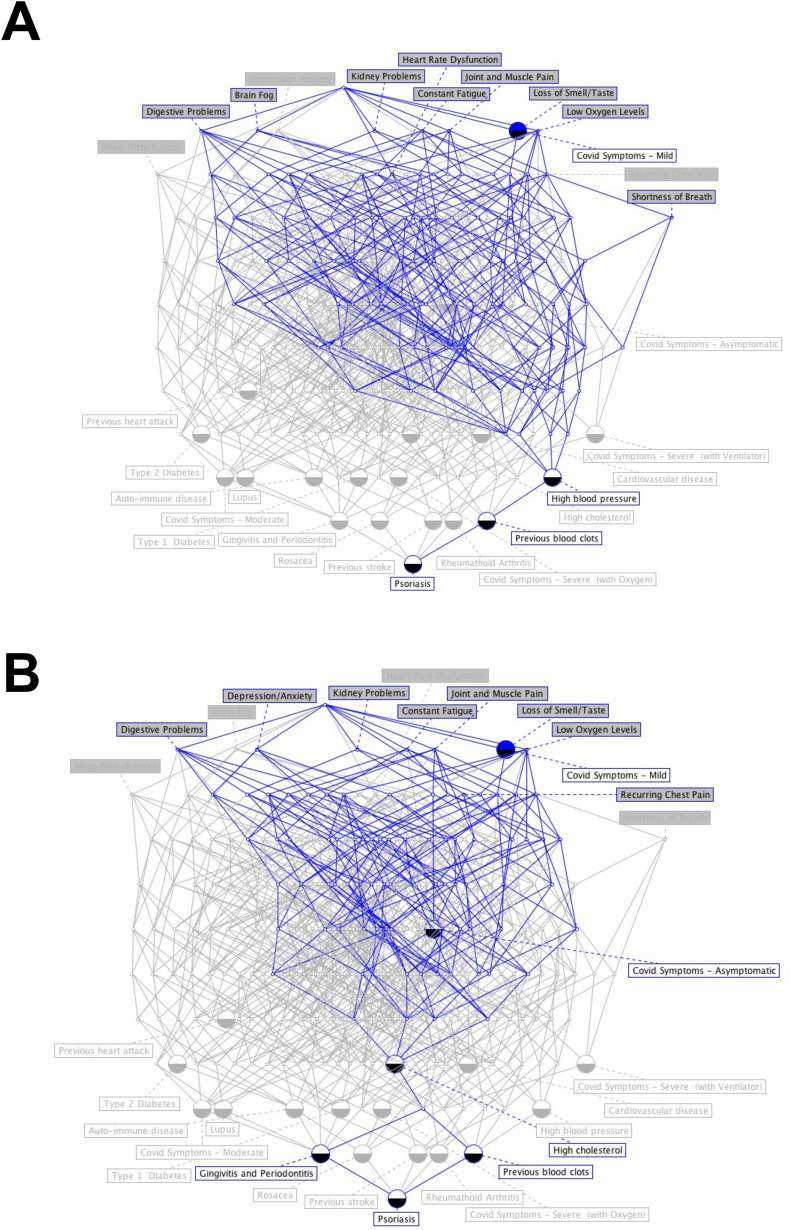
**A, B** Lattice plots, showing participants comorbidities versus Long COVID/PASC symptoms lattice, highlighting the high blood pressure and high cholesterol comorbidities

**Fig. 6:**
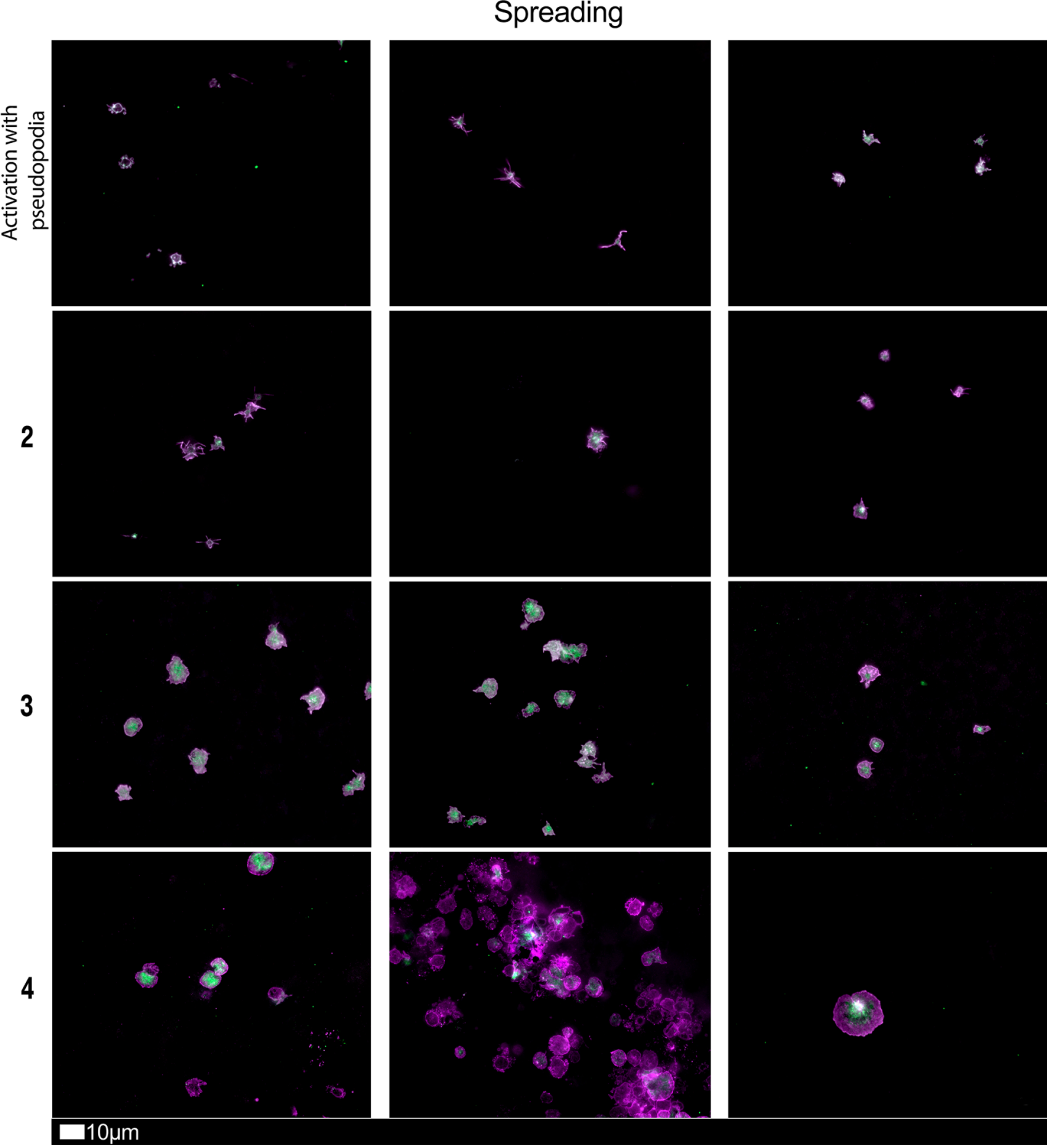
Fluorescence microscopy examples of the different stages of platelet activation and spreading, that was used to score the platelet activation in the Long COVID patients, with Stage 1, with minimally activated platelets, seen as small round platelets with a few pseudopodia, seen as healthy/control platelets that progresses to Stage 4, with egg-shaped platelets, are indicative of spreading and the beginning of clumping. Taken from [28] with permission

In Figs. 2, 3, 4, 5 and 6, the distribution of the South African Long COVID/PASC registry participant data (845 participants) was analysed according to the patients’ gender, comorbidities, age group, initial COVID-19 symptoms, and Long COVID/PASC symptoms, using Sankey plots. The same participant versus comorbidity versus symptom data were further manipulated to produce a mapping between comorbidities and symptoms, represented as a matrix with comorbidities as rows and symptoms as columns. This was used to draw a lattice, giving insight into the implications (simple binary implications for visualisation) from comorbidities to symptoms. The corresponding lattices with different components highlighted, correspond to the most prominent comorbidities emerging from the Sankey diagrams: high blood pressure, high cholesterol, Type 2 diabetes, auto-immune disease, and previous blood clots. In the following figures (Figs. 1, 2, 3, 4 and 5), we rehearse the implications in more detail.

Figure 1 gives a general overview of the South African Long COVID/PASC registry. About 10% (i.e. 87) of the participants were not initially tested for SARS-CoV2 using a PCR test, whereas in 90% (i.e. 758) of the patients, a COVID-19 positive test was reported. Moreover, patients were also categorised according to gender. Thus, 70% and 30% (i.e. 593 and 252) of the study cohort were identified as female and male, respectively, in line with common observations [34, 35]. The majority (i.e. 76%) of the participants were between the ages of 31–40, 41–50, and 51–60 years. We observed that participants with comorbidities such as high blood pressure, high cholesterol, type-2 diabetes, autoimmune disease, and previous blood clots were in the majority.

Figure 2 shows the gender distribution for the participants with the Long COVID/PASC symptoms in more detail. In a similar trend as observed in Fig. 2, the common Long COVID/PASC symptoms were noted as fatigue; brain fog, loss of concentration, and forgetfulness; shortness of breath, as well as joint and muscle pains. Interestingly, Long COVID/PASC symptoms such as kidney problems, digestive problems, and low oxygen levels were less commonly reported. Figure 3 shows the age versus Long COVID/PASC symptoms distribution of the participants. We note that the majority of participants were within the age ranges of 31–40, 41–50, and 51–60 years.

Figure 4 shows a Sankey plot that illustrates the population distribution of participants’ comorbidities versus Long COVID/PASC symptoms, while Fig. 5A, B shows representative lattice plots of high blood pressure and high cholesterol levels, confirming in more detail the correlations already shown in Figs. 3 and 6. Reading upwards in the lattice, for example in 8A, the “high blood pressure” node connects upwards through a highlighted network to a variety of symptoms, ranging from “digestive problems” (on the left) to “shortness of breath” on the right. The complexity/density of the blue highlighted network represents prevalence of the comorbidity amongst the patients, as well as implications of a variety of symptoms.

### Blood analysis

**Fig. 7:**
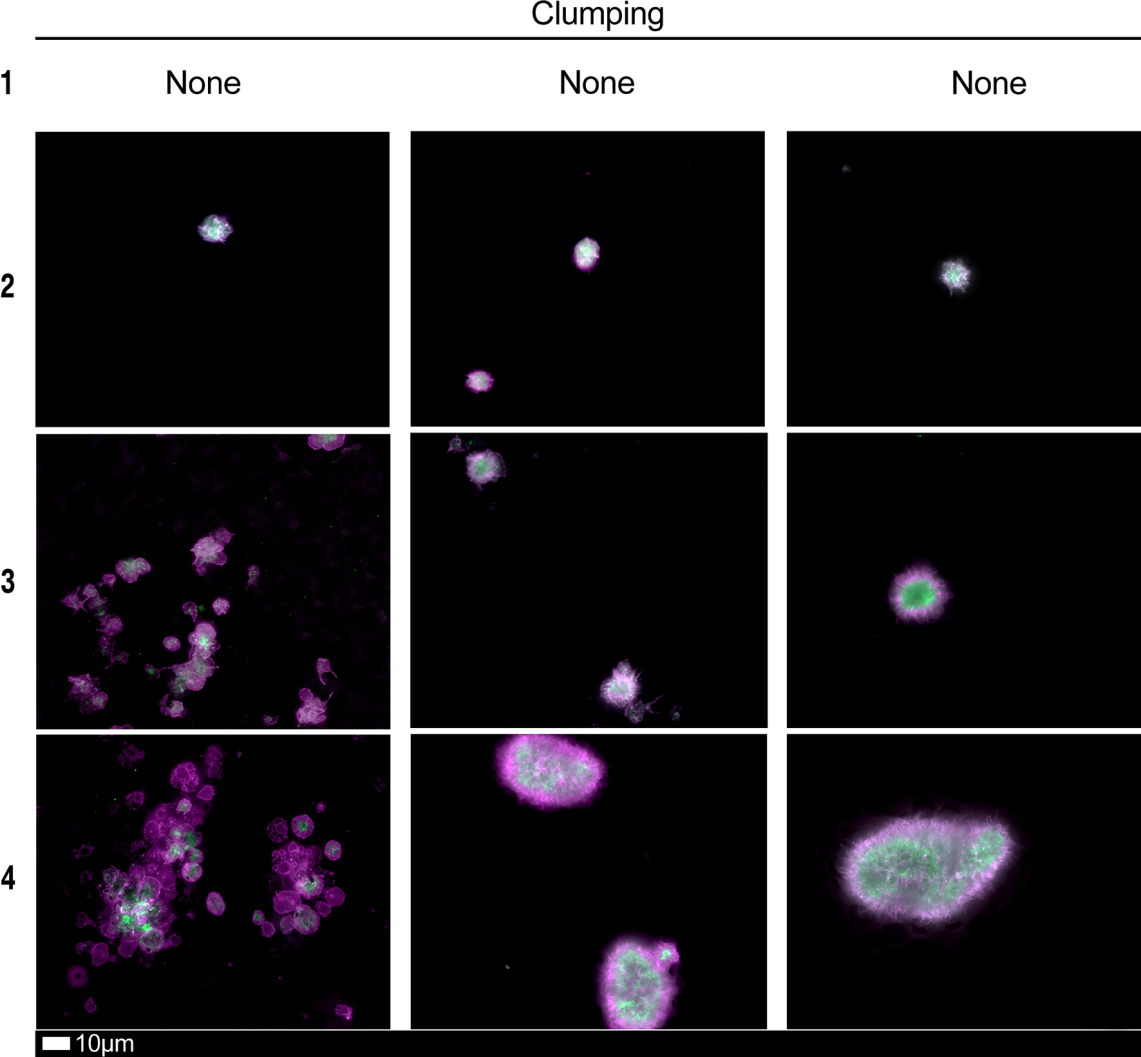
Fluorescence microscopy examples of the different stages of platelet clumping. With no clumping occurring in the healthy/control samples in Stage 1 (no figures shown), progressing to severe clumping of platelets as seen in Stage 4. Taken from [28] with permission

We studied blood samples from 80 diagnosed Long COVID/PASC patients (age median/SD 48 ± 16) (35 females and 45 males). Microclot and platelet analysis showed presence of microclots and platelet pathologies in all 80 patients. We used a platelet grading system to identify platelet pathologies, that we have developed and described previously [28] see Figs. 6 and 7 and Table 1. Platelet and microclot grading were done on a subset of 30 of these individuals.

**Table 1:** Platelet activation criteria showing the level of spreading, as well as clumping in the haematocrit sample. Taken from [28] with permission

Score	Spreading	Score	Clumping
1	Activation with pseudopodia	1	None
2	Mild	2	Mild
3	Moderate	3	Moderate
4	Severe	4	Severe

**Fig. 8:**
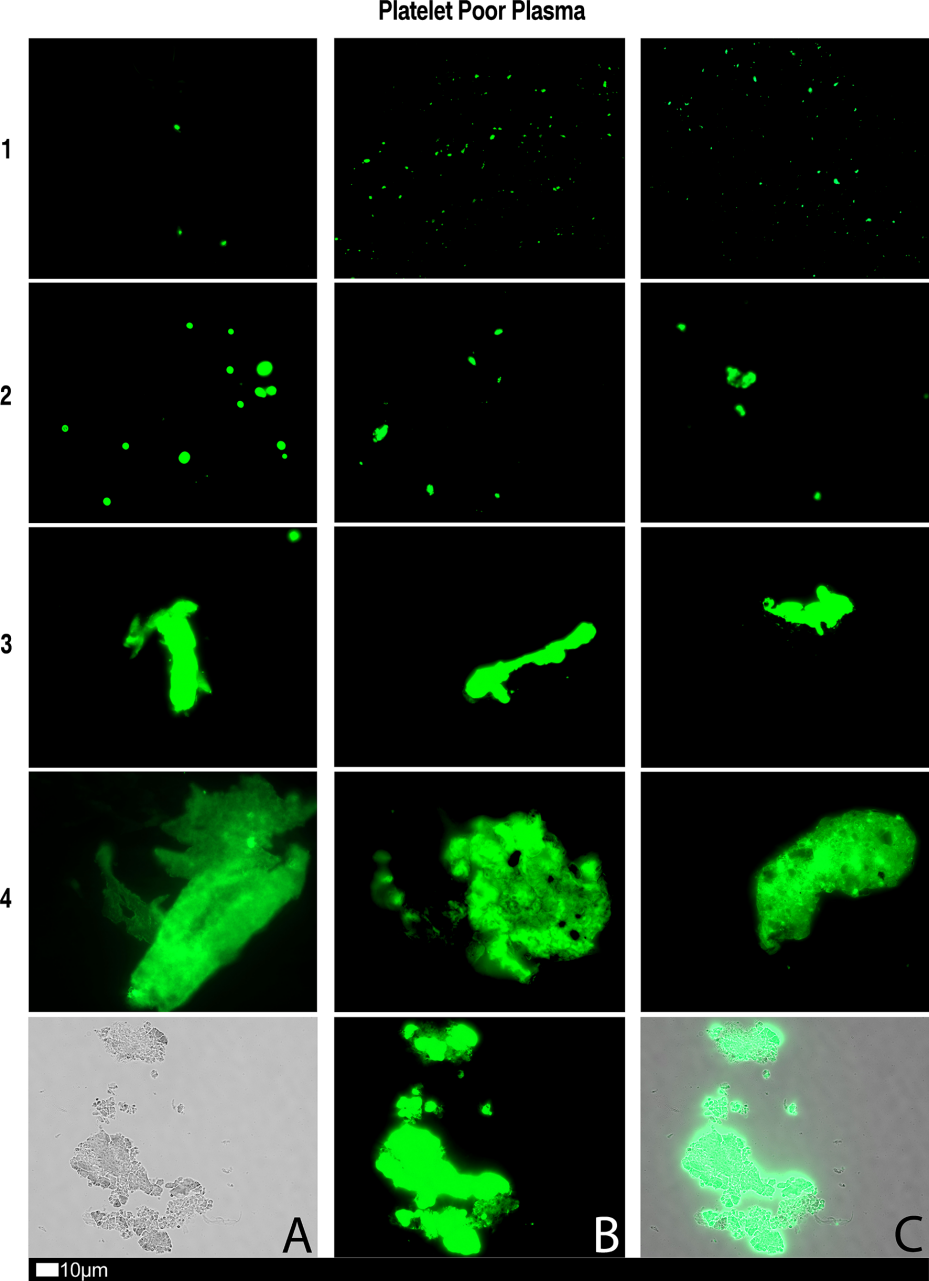
Fluorescence microscopy showing microclots in platelet poor plasma (PPP) with representative examples of the different stages microclot formation. Stage 1 shows minimal microclot formation in healthy/control PPP which progresses to the presence of the severe microclotting Stage 4. The bottom row represents examples of stage 4 microclots using **A** bright-field microscopy, **B** fluorescence microscopy, and C an overlay of fluorescence and bright-field microscopy. Taken from [28] with permission

We also used a clotting grading system that we have developed and have published previously [28], see Fig. 8 and Table 2. Both the scoring of the platelet pathology and PPP microclots were combined and given a final score to determine the severity of the disease (Table 3).

**Table 2:** Microclot criteria to determine the amount of microclots in the platelet poor plasma sample. Taken from [28] with permission

Score	Analysis criteria
1	Very few areas of plasma protein misfolding (≤ 1 µm) visible with a few ≤ 10 µm microclots
2	Very few areas of plasma protein misfolding (≤ 1 µm) visible with scattered/mild ≤ 10 µm microclots
3	Moderate areas of plasma protein misfolding visible as microclots ≥ 15 µm
4	Severe areas of plasma protein misfolding visible as large microclots

**Table 3:** Overall microclot and platelet activation score results.

Scoring results			
Control/healthy	Mild	Moderate	Severe
= 3	4–7	8–10	11–12

Our overall platelet and microclot scoring results for 30 of the 80 patients were 7 ± 1.3, pointing to moderate activation.

**Fig. 9:**
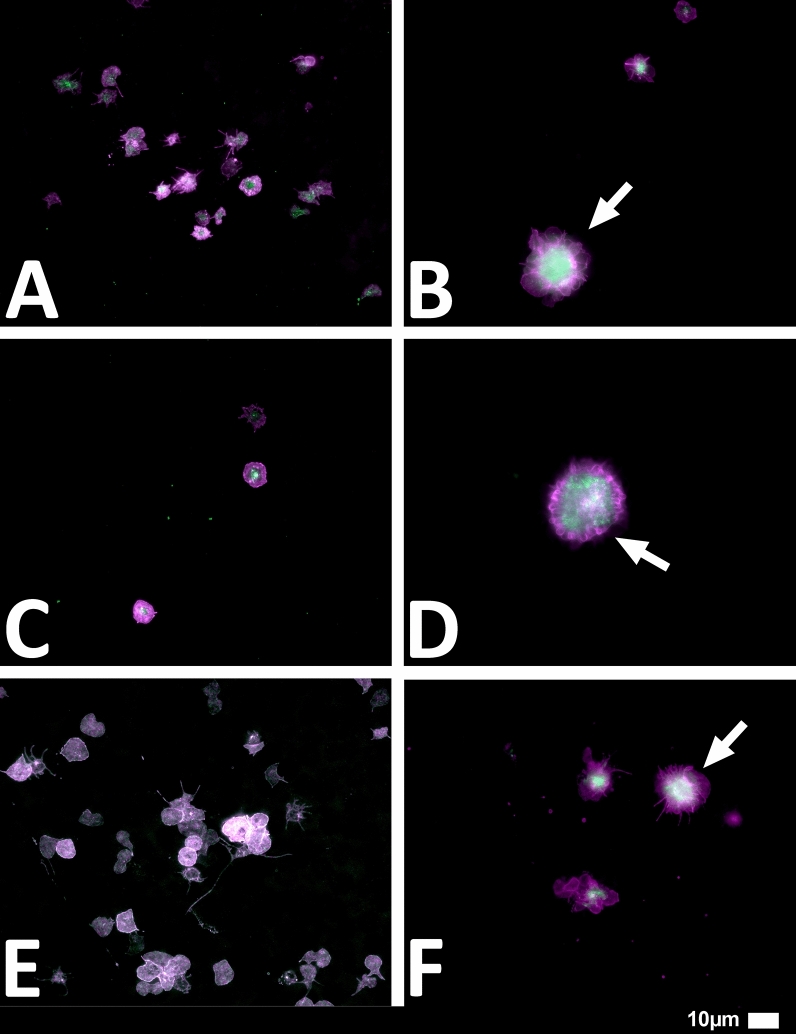
Representative micrographs of hyperactivated platelets in individuals with Long COVID/PASC, with the arrows indicating mild platelet clumping

**Fig. 10:**
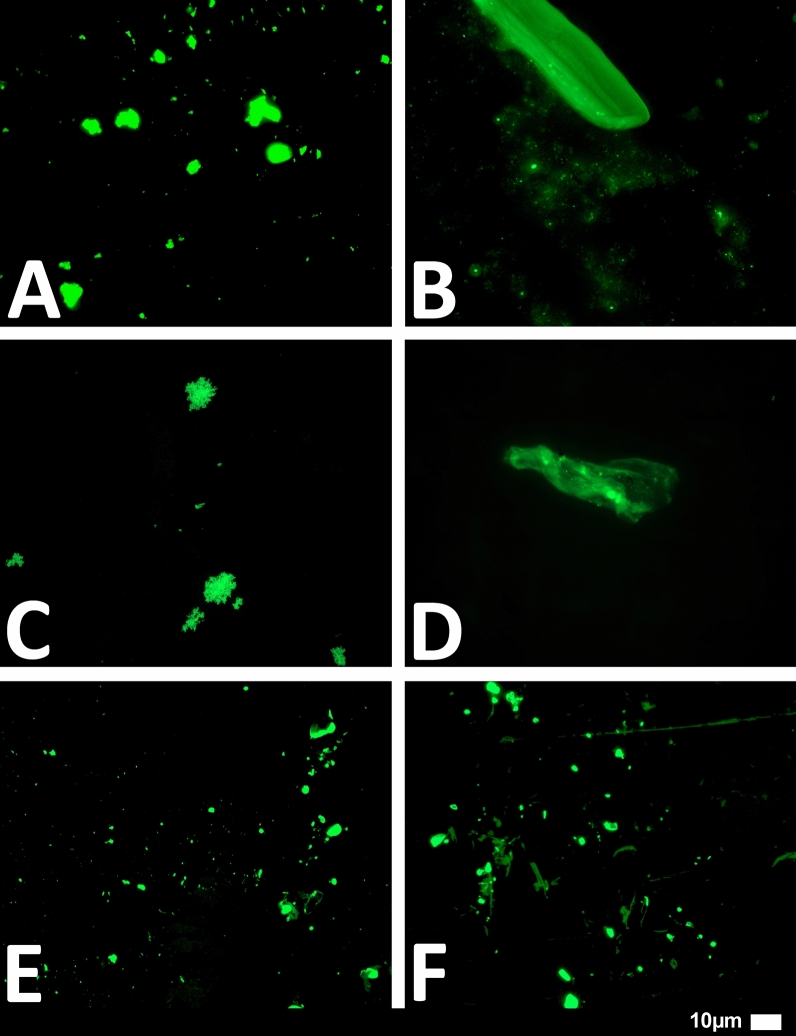
Representative micrographs of microclots in individuals with Long COVID/PASC. Moderate plasma microclots can be seen in the individuals with Long COVID/PASC

**Fig. 11:**
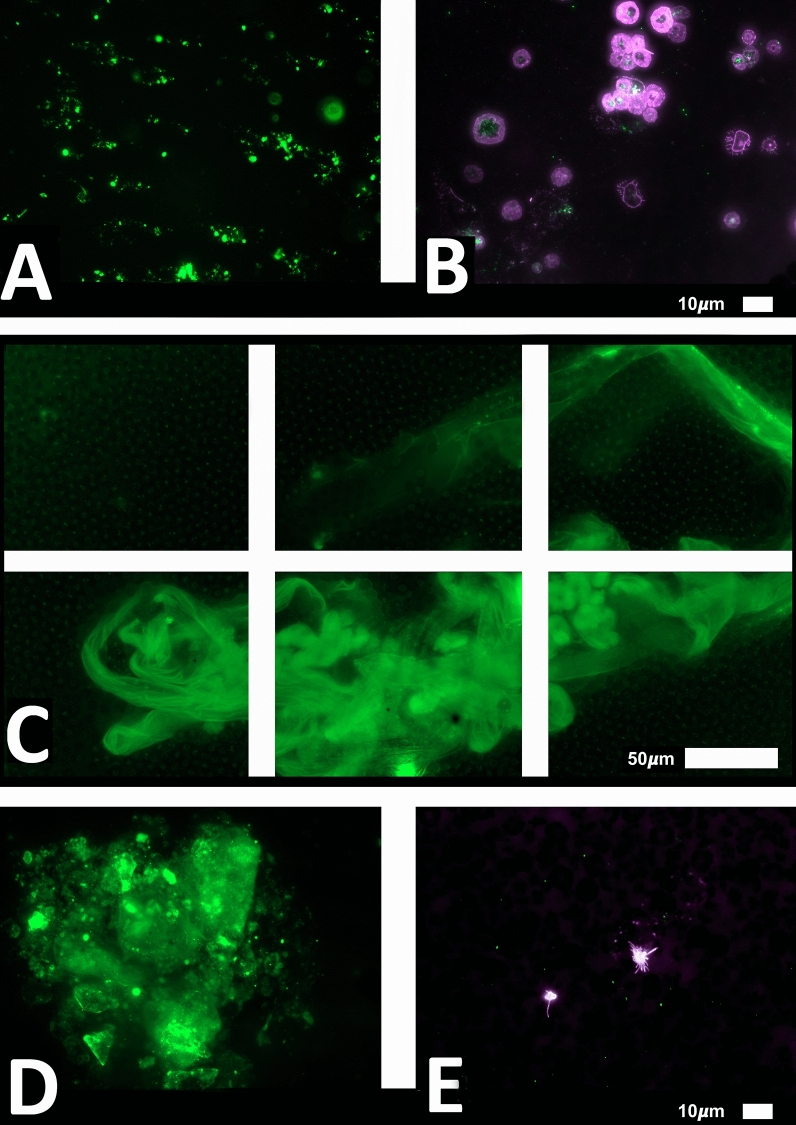
Microclots and platelets in two individuals with Long COVID/PASC. **A** Representative micrographs of microclots in an untreated patient diagnosed with Long COVID/PASC, suffering from the condition for 11 months. The plasma was stained with thioflavin T (ThT); **B** Platelet hyperactivation of the same patient, where PAC-1 and CD62PE were used to mark platelets. **C** In this patient, cellular debris in the haematocrit was noted—here such cellular debris is shown in a tile scan. **D**,** E** Microclot presence and platelets from a patient who was on self-administrated aspirin (anti-platelet) treatment before blood collection, where major microclots were noted, but the platelets were minimal hyperactivated, possibly due to the use of anti-platelet therapy

Figures 9 and 10 show representative micrographs of platelet activation and the presence of fibrin amyloid microclots in two of the Long Covid/PASC participants blood samples. Figure 11 shows representative micrographs of a patient suffering from Long COVID/PASC for 11 months, where Fig. 11A, B show a tile scan of cellular debris present in the haematocrit. Figure 11D, E show representative microclots and platelets where the patients have been using Aspirin only, before sample collection. As anticipated, significant microclot formation were still seen, but platelets were not significantly activated.

## Discussion

Here we report on the comorbidities and symptoms identified in a cohort of 845 South African Long COVID/PASC patients who filled in the South African Long COVID/PASC registry. We show that hypertension, high cholesterol levels (dyslipidaemia) and T2DM are important comorbidities that may play a significant role in the development of Long COVID/PASC in this cohort. (We also recognise that other comorbidities, such as previous viral infections, are also very important, but may manifest only in a larger cohort). It is well-documented that impaired endothelial function is associated with increased cholesterol and hypertension (which may be underpinned by generic variation) due to increased vascular oxidative stress and inflammation [36, 37]. Here we also showed the presence of fibrin amyloid microclotting and platelet pathologies in another cohort of 80 patients that visited a clinical practice complaining of persistent symptoms, where these patients were diagnosed with Long COVID/PASC. We found that in this cohort, all of the patients did indeed have both increased amyloid microclotting, as well as platelet pathologies, as assessed by a platelet and clotting grading system that we have developed previously [28].

Normal blood clotting goes through a variety of established mechanisms [33], a major step being the cleavage by thrombin of the complex fibrinogen molecule (roughly cylindrical, with a 5 × 45 nm size). This releases two fibrinopeptides, and causes the thermodynamically favourable formation of fibrin macrofibres, typically 50–100 nm in diameter. They may be crosslinked by Factor XIII. The clots are usually removed by fibrinolysis, leading to the residual formation of d-dimer; its normally low background levels reflect this background activity. It was always assumed that the normal conformation of a protein is that of its lowest free energy, as per Christian Anfinsen’s famous protein refolding experiments. However, this is not the case. Many proteins can fold into a form of lower free energy but retain the identical sequence. Some of these forms, containing ordered beta-sheet structures, are generically referred to as amyloids, and many are well known to be associated with certain diseases. Ab in Alzheimer’s disease and synuclein in Parkinson’s disease are examples, with over 50 recognised [20]. Note, however, that almost any protein can form amyloid structures (e.g. recombinant insulin will do it over time, as will lysozyme held at an acid pH). Another well-known example of a class of proteins that can exist in two conformations of identical sequence is represented by prion proteins. The normal form with alpha-helices is called PrP^c^ and the amyloid one PrP^Sc^, the latter being of lower free energy i.e. more thermodynamically stable. It is also highly resistant to proteolysis. This transition between the two forms can itself be catalysed by the PrP^Sc^. The key point of importance in microclot formation in Long COVID/PASC is that fibrin(ogen) too can, in the presence of various trigger substances, fold into an amyloid form that has a very different macrostructure characterised by different fibre diameters and pore sizes [38]. We noted for example that in type 2 Diabetes Mellitus (T2DM) clots have a netlike appearance [39–42], while in Alzheimer’s disease [18, 43–45] and Parkinson’s disease the fibres may be larger in size [17, 24]; they are also much more resistant to proteolysis [19], and are much more prevalent in the steady state.

We have been observing fibrin(ogen) changes generally for many years, initially via electron microscopy (e.g. [46–51], and many others). Since 2011 we have also studied the effect of fibrin(ogen) folding in the presence of various inflammatory molecules, such as iron ions [52], that can stimulate the anomalous fibrin form [53]. These anomalous structures could also be found in a variety of disease states [19], such as T2DM [40] and Alzheimer’s disease [54]. In this earlier literature we often referred to these anomalous clots as ‘dense matted deposits’. In 2016, we showed that this anomalous resistance to fibrinolysis was because the anomalous structures were in fact amyloid in nature [38]. Such structures are easily observed under the optical microscope, and in particular may be stained with the fluorogenic dye thioflavin T and by the more recently developed oligothiophene dyes marketed by Ebba Biotech as Amytrackers [17, 21, 23].

**Fig. 12:**
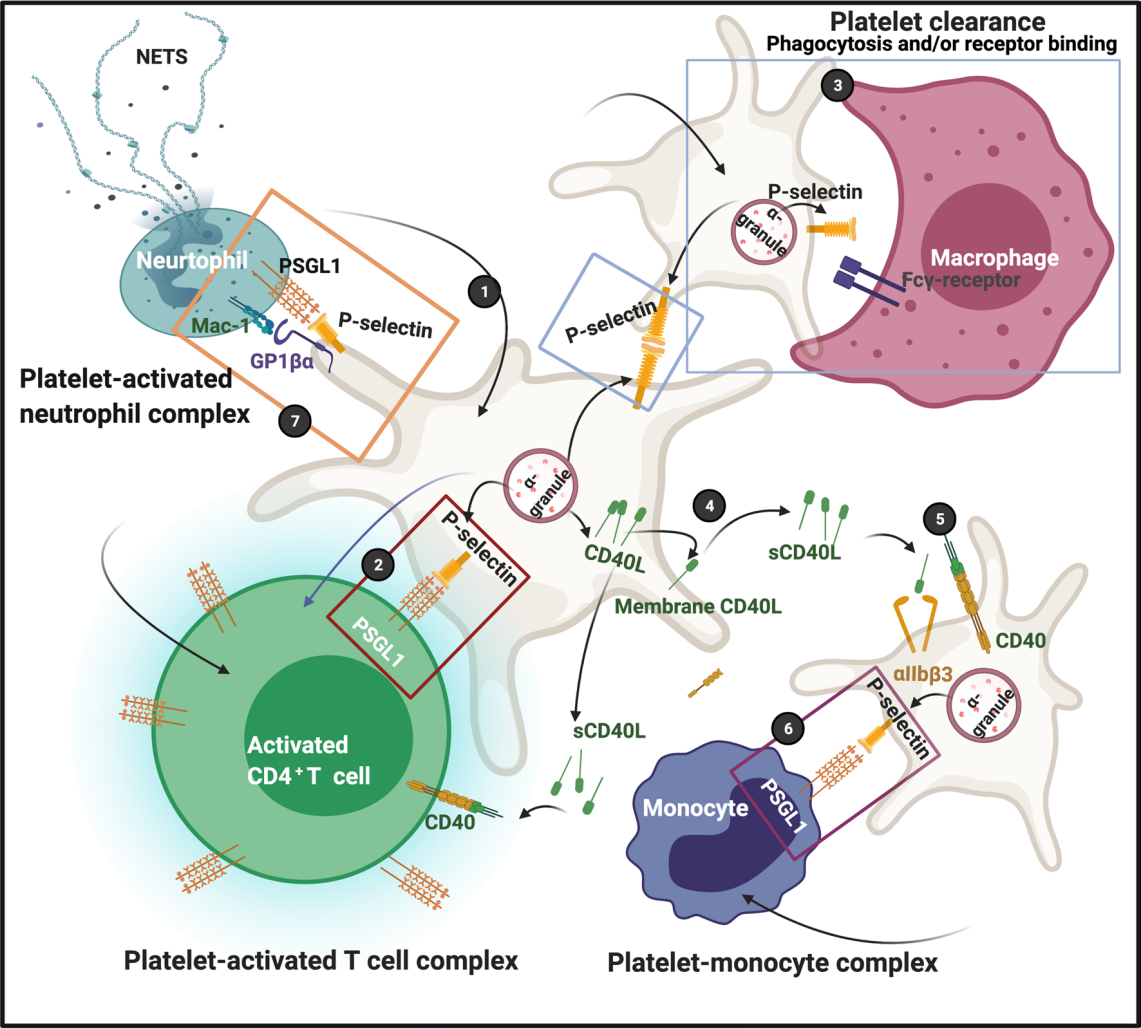
(**1**) After activation, platelets express P-selectin on their membranes, followed by platelet-T cell complex formation (**2**); P-selectin on platelet membranes are also recognized by macrophages, possibly by the Fcγ-receptor; clearance may result due to either receptor binding or phagocytosis (**3**). CD40L is released from platelets and can migrate to membranes or shed as soluble (s)CD40L (**4**). sCD40L can bind to both the α_IIb_β_3_ or CD40 receptors (**5**). The P-selectin on the membranes of sCD40L-activated platelets can also form complexes with monocytes (**6**). Platelet-neutrophils also form complexes (**7**). Diagram created with BioRender (https://biorender.com/) and adapted from [55]

Most recently, we have demonstrated this explicitly in COVID-19 patients [12, 13]. Here, these microclots were observed without the addition of clotting agents, i.e. thrombin, and therefore, these amyloid microclots were in the plasma of the individuals at the time of sampling. In particular, it was found not only that the clots contained fibrin (fibrinogen being one of the most concentrated proteins in plasma), but that these microclots had entrapped alpha-2-antiplasmin [15] and a variety of other proteins and even antibodies, which therefore were not observed in plasma from which the microclots had been removed (so did not appear as biomarkers, even though they were present). Because these clots are insoluble and effectively inert, they do not contribute to plasma viscosity as determined via TEG®, whose values can thus appear normal in Long COVID/PASC (unpublished data). Another characteristic of COVID-19 is the extremely high levels of activation of platelets [13]. Together with platelet pathology and the presence of microclots in the circulation, endothelial damage may be key drivers of persistent Long COVID/PASC symptoms [15]. See Fig. 12 for a snapshot of the interactions that platelets have with circulating blood cells and the various complexes they form (for a detailed review, see [55]).

## Conclusion

In the current study, we report data from the South African Long COVID Registry for the first time. It was noted that each of 80 patients diagnosed with Long COVID/PASC and who provided blood samples, showed platelet hyperactivation and microclot formation. We suggest that a platelet and clotting grading system should be used as a simple and cost-effective diagnostic method for the early identification of Long COVID/PASC. Diagnosis of Long COVID/PASC requires exclusion of other pathologies and evaluation of the duration of symptoms (> 2 months after acute infection). If a bleeding tendency (not seen commonly) is a concern, a TEG® can be used as a safety-net to not overtreat the patient. The exact combination of treatment and duration needs further investigation and testing in randomized control trials. Unfortunately, treatment protocols are not yet widely available and current protocols are based on clinician-initiated approaches and experience in managing these patients. No clinical trials have been performed yet; however, this will be an important next step to urgently consider in parallel to patient monitoring using a multi-modal pathology-supported genetic testing approach [56]. For implementation of personalised medicine it will be essential to bring together clinicians, researchers, patients and policy makers to advance healthcare while allowing for adjustment and flexibility in view of new discoveries [57]. The large number of affected individuals who develop Long COVID has major detrimental effects on public health and necessitates long-term follow-up and support that can be mediated via the Long COVID Registry as a major strength of this study.

## Data Availability

The raw data supporting the conclusions of this article will be made available by the authors, without undue reservation.
